# Temperature Measurements in the Vicinity of Human Intracranial EEG Electrodes Exposed to Body-Coil RF for MRI at 1.5T

**DOI:** 10.3389/fnins.2020.00429

**Published:** 2020-05-12

**Authors:** Hassan B. Hawsawi, Anastasia Papadaki, John S. Thornton, David W. Carmichael, Louis Lemieux

**Affiliations:** ^1^Department of Clinical and Experimental Epilepsy, UCL Queen Square Institute of Neurology, London, United Kingdom; ^2^MRI Unit, Epilepsy Society, Buckinghamshire, United Kingdom; ^3^Administartion of Medical Physics, King Abdullah Medical City, Makkah, Saudi Arabia; ^4^Lysholm Department of Neuroradiology, National Hospital for Neurology and Neurosurgery, UCLH NHS Foundation Trust, Queen Square, London, United Kingdom; ^5^Department of Brain Repair and Rehabilitation, UCL Queen Square Institute of Neurology, London, United Kingdom; ^6^Developmental Imaging and Biophysics Section, UCL Great Ormond Street Institute of Child Health, London, United Kingdom; ^7^Wellcome EPSRC Centre for Medical Engineering, King’s College London, St Thomas’ Hospital, London, United Kingdom

**Keywords:** safety, intracranial, EEG, MRI, body transmit coil, icEEG-fMRI, RF heating

## Abstract

The application of intracranial electroencephalography (icEEG) recording during functional magnetic resonance imaging (icEEG-fMRI) has allowed the study of the hemodynamic correlates of epileptic activity and of the neurophysiological basis of the blood oxygen level-dependent (BOLD) signal. However, the applicability of this technique is affected by data quality issues such as signal drop out in the vicinity of the implanted electrodes. In our center we have limited the technique to a quadrature head transmit and receive RF coil following the results of a safety evaluation. The purpose of this study is to gather further safety-related evidence for performing icEEG-fMRI using a body RF-transmit coil, to allow the greater flexibility afforded by the use of modern, high-density receive arrays, and therefore parallel imaging with benefits such as reduced signal drop-out and distortion artifact. Specifically, we performed a set of empirical temperature measurements on a 1.5T Siemens Avanto MRI scanner with the body RF-transmit coil in a range of electrode and connector cable configurations. The observed RF-induced heating during a high-SAR sequence was maximum in the immediate vicinity of a depth electrode located along the scanner’s central axis (range: 0.2–2.4°C) and below 0.5°C at the other electrodes. Also for the high-SAR sequence, we observed excessive RF-related heating in connection cable configurations that deviate from our recommended setup. For the low-SAR sequence, the maximum observed temperature increase across all configurations was 0.3°C. This provides good evidence to allow simultaneous icEEG-fMRI to be performed utilizing the body transmit coil on the 1.5T Siemens Avanto MRI scanner at our center with acceptable additional risk by following a well-defined protocol.

## Introduction

Intracranial electroencephalography during functional magnetic resonance imaging (IcEEG-fMRI) has been used to map epileptic activities (Vulliemoz et al., 2011; Boucousis et al., 2012; Carmichael et al., 2012) with much greater sensitivity enabling more detailed, quantitative studies of interictal, preictal and ictal epileptogenic networks (Vulliemoz et al., 2011; Cunningham et al., 2012; Aghakhani et al., 2015; Beers et al., 2015; Chaudhary et al., 2016; Ridley et al., 2017; Sharma et al., 2019) and of neuronal events more generally (Murta et al., 2016, 2017; Saignavongs et al., 2016).

However, simultaneous icEEG-fMRI is prone to signal loss around the icEEG electrodes and more particularly when using echo-planar imaging (EPI) sequences due to magnetic susceptibility effects; using gradient echo EPI we found up to 50% signal drop at around 5 mm from the electrode contacts (Carmichael et al., 2012). Currently, our icEEG-fMRI acquisitions are limited to the head transmit and receive RF coil, in accordance with the conclusions of our previous investigations on the technique’s feasibility (Carmichael et al., 2008, 2010, 2012). The use of the body transmit coil in conjunction with the use of a head receive coil array would allow the use of parallel imaging techniques to reduce scanning time and susceptibility effects (Pruessmann et al., 1999; Larkman et al., 2001; Griswold et al., 2002; Setsompop et al., 2012).

In terms of subject safety, the combination of icEEG-fMRI constitutes a particularly challenging imaging technique due to a number of health risks (in addition to the invasiveness of icEEG electrode placement), associated with the exposure of metallic implants to the three fields used in MRI, namely: static magnetic field (B_0_), the radiofrequency (RF) field (B_1_) and the switching gradient magnetic fields. In principle, the B_0_ field can cause an implant to experience a net force (displacement) or rotational (torque), the RF field can result in heating of the tissues around the implants and the gradient fields can induce eddy currents resulting in neural stimulation (Carmichael et al., 2010; Hawsawi et al., 2017). The exhaustive safety and data quality tests (Carmichael et al., 2008, 2010, 2012) that preceded our implementation of icEEG-fMRI lead us to define a data acquisition protocol that limits us to use a head RF-transmit/receive coil [in addition to low SAR sequences and positioning of the electrode wires along the RF coil’s central (Z) axis], with important implications for blood oxygen level-dependent (BOLD) sensitivity (Carmichael et al., 2012).

In view of further developing icEEG-fMRI by modifying our protocol to allow the use of our MRI scanner’s body transmit RF coil, we undertook new phantom tests to assess the conditions under which the body RF-transmit coil could be used with an acceptable level of additional risk. This article focuses on characterizing the RF-induced heating in the vicinity of icEEG electrodes exposed to RF produced by our 1.5T MRI scanner’s body transmit coil with different lead configurations and whether these were connected to the recording system or not.

## Materials and Methods

**FIGURE 1:**
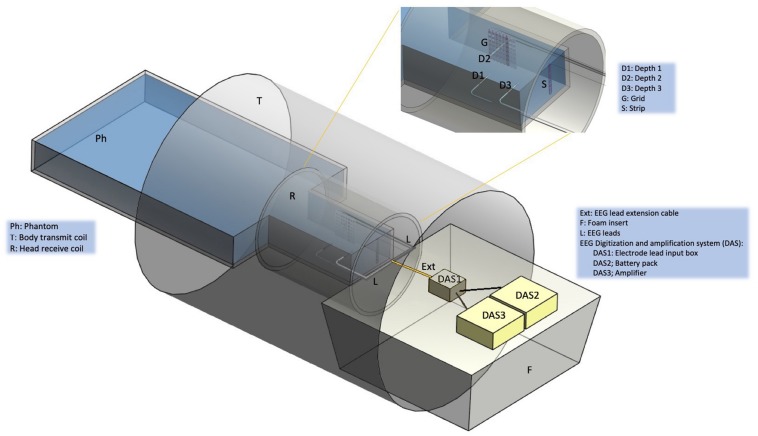
Experimental set up. Schematic representation of the gel-filled phantom (Ph), scanner RF coils (T and R), foam insert (F) and EEG electrodes, leads and digitization and amplification system (DAS). The following icEEG electrode were placed in the gel-filled phantom: *Depth 1* (D1) orientated laterally, *Depth 2* (D2) (lateral), *Depth 3* (D3) (lateral), *Grid* (G) (para-sagittal), *Strip* (S) (anterior-posterior). Depending on the experimental configuration (see Figure 2), the electrodes were connected to leads (L), extension cables (Ext), and the DAS system [electrode lead input box (DAS1), battery pack (DAS2), amplifier (DAS3)]. Depending on the experimental configuration, the DAS system was placed either on top of a foam insert (F) or at the bottom of the scanner bore (no foam insert).

We measured RF-induced temperature changes in the immediate vicinity of icEEG electrodes placed in a standard test phantom exposed to a body transmit coil, over a range of lead placement and termination configurations. In line with our previous work on the safety of icEEG-fMRI (Carmichael et al., 2008, 2010) five icEEG electrodes were placed in the head part of the phantom to simulate a representative, realistic clinical scenario (see Figure 1).

### Phantom Preparation

Following the ASTM F-2182-02a guidelines, we used a container made of acrylic with the following dimensions: head length = 290 mm, head width = 195 mm, torso length = 300 mm, torso width = 330 mm and height = 150 mm (Figure 1). It was filled with 0.70 g/L of NaCl, 8 g/L of polyacrylic acid (PAA) and 15 L of distilled water in order to simulate human brain tissue electrical conductivity of 0.26 S/m (Park et al., 2003).

### EEG Electrode, Connecting Lead and Recording System Configurations

Three depth icEEG electrodes, two 8–contact (Ad-Tech model SD08R-SP10X-000; 8 platinum contacts, 10 mm spacing, 72 mm recording area and 380 mm total depth length) and one 10-contact (Ad-Tech model SD10R-SP10X-000, 10 platinum contacts, 10 mm spacing, 92 mm recording area, and 390 mm total depth length) were positioned as follows, along lateral trajectories mimicking a bilateral mesial temporal lobe implantation: *Depth 1* with eight contacts was positioned on the left hand side [9.86 cm from the superior aspect of the phantom (top of the head) and 5.5 cm from the anterior surface (face; depth from the gel’s surface), with the deepest contact located 12 cm from the left lateral surface]; similarly, *Depth 2* with eight contacts was inserted (through a hole in the *Grid* electrode – see below) on the right hand side [9.28 cm from the superior aspect of the phantom (top of the head) and 5.5 cm from the anterior aspect (face; depth from the gel’s surface), and the depth of 7.5 cm from the right lateral surface]; and *Depth 3* with 10 contacts in the left hand side located 10 mm superior to *Depth 1* in the same coronal plane and depth. A *Grid* electrode (Ad-Tech model FG64D-SS10X-0E2, 10 mm spacing, 64 platinum contacts, nichrome wire, and electrode total length of 455 mm) was placed in a para-sagittal plane in a location to emulate the placement of electrodes over the left cortical region and located 2 cm away from the head’s lateral aspect. A *Strip* electrode (Ad-Tech model TS06R-AP10X-0W6, 6 platinum contacts, 10 mm spacing, 72 mm recording area and 380 mm total depth length) was located in an axial plane in the superior part of the phantom head (2.18 cm from the top of the head).

Lead extension wires (length = 90 cm), which are used to connect the electrode leads to the EEG digitization and amplification system [DAS; consisting of the electrode lead input box, battery pack and amplifier(s)] for the purpose of recording, were used in some of the heating tests.

Following our routine practice for patient scanning sand bags were placed on top of the electrode leads and cables along their path from the phantom to the DAS (Vulliemoz et al., 2011). In accordance with our icEEG-fMRI data acquisition protocol (Carmichael et al., 2012) a MRI scanner EEG equipment positioning foam insert manufactured by us, to be placed at the head end of the scanner bore (between the head coil and bore opening at the scanner far end) was used in the tests to ensure the reproducible and secure placement of the electrode lead tails and extensions, and EEG DAS in the scanner bore (Figure 1; Carmichael et al., 2012). The positioning foam insert consists of a hemi-cylinder (length: 79.7 cm) with a radius that matches the scanner bore’s internal diameter, and has grooves and cut outs (depth: 0.8 cm) in its (top) flat surface to enable reproducible placement of the leads and DAS along the scanner’s central (Z) axis, to minimize the coupling between the EEG system and RF *E* field, which by design is made to have the smallest possible magnitude on the Z-axis within the scanning field of view (Lemieux et al., 1997). In some of the tests described below, the effect of not using the positioning foam insert on the RF-induced heating was assessed; without the foam insert in place, the leads and EEG DAS rest on the bottom of the scanner bore (therefore away from the Z-axis, closer to the body coil).

Our previous work has demonstrated the effects of electrode and lead placement, and of electrical termination on the amount of RF-induced heating in the vicinity of icEEG electrodes (Carmichael et al., 2008, 2010). Two sets of measurements were performed: *Experiment 1* and *Experiment 2*; each set corresponding to a scanning session, and designed to provide an assessment of heating increases in the tissue surrounding the icEEG electrodes using different lead configurations for the body RF transmit coil, and assessing reproducibility by repeating some measurements. The configurations are labeled *A(i)* (*i* = 1, 2, 3) for *Experiment 1* and *B(i)* (*i* = 1,…, 5) for *Experiment 2*.

#### Experiment 1

In this experiment, we set out to perform an evaluation of the effects of using the body transmit RF coil on the heating in the vicinity of icEEG (*Depth 1*, *Depth 2*, *Depth 3*, *Strip*, and *Grid*) electrodes located inside a water-based phantom. Previous studies (Carmichael et al., 2008, 2010; Boucousis et al., 2012; Ciumas et al., 2013) studied the effect of body transmit coil and concluded that body transmit coil produces significant temperature increase above the safety levels, and we sought to update this information for the configurations of electrodes, connecting leads and EEG DAS specified in our successfully implemented protocol (Carmichael et al., 2012). We also sought to explore slight variations on this arrangement and to obtain temperature measurement reproducibility data.

**FIGURE 2:**
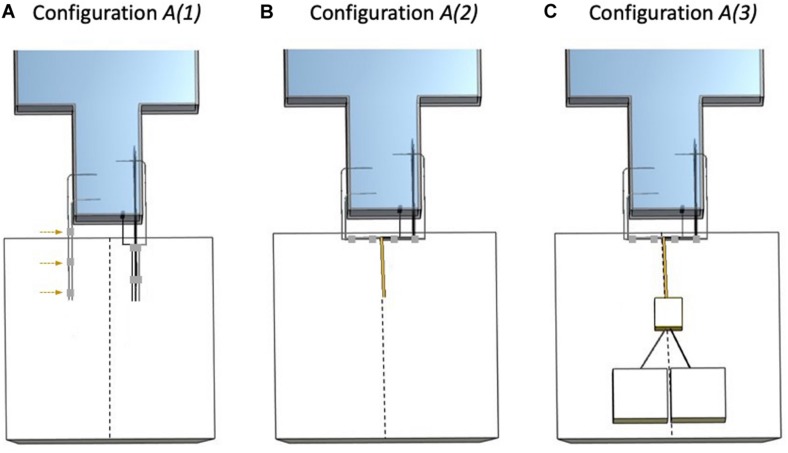
Electrode configurations A (*Experiment 1*). The dashed line represents the scanner’s central Z-axis as drawn on the foam insert. **(A)** Configuration *A(1)*: *Strip*, *Grid*, and *Depth 2* bundled together (right bundle) and separated from the left bundle (*Depth 1* and *Depth 3*) without lead extensions and with lead tails placed on top of the foam insert. The adhesive tape (gray) used to create the left bundle is highlighted with the dashed arrows. **(B)** Configuration *A(2)*: *Strip*, *Grid*, and *Depth 2* bundled together (right bundle), connected to 90 cm lead extensions and placed on top of the foam insert. *Depth 1* and *Depth 3* bundled together (left bundle) and unterminated with lead tails on top of the foam insert. **(C)** Configuration *A(3)*: *Strip*, *Grid*, and *Depth 2* bundled together (right bundle), connected and terminated by connection to the EEG DAS using the 90 cm lead extensions, placed on top of the foam insert. *Depth 1* and *Depth 3* bundled together (left bundle) and unterminated with lead tails on top of the foam insert.

The three following electrode configurations were studied in *Experiment 1* (see Figure 2):

***A(1)***: With foam insert. No lead extensions. Electrodes unterminated with *Strip*, *Grid*, and *Depth 2* lead tails bundled together (using adhesive tape) on the right side of the superior aspect of the phantom head (right bundle) and *Depth 1* and *Depth 3* lead tails bundled together on the left (left bundle) and separated from the right bundle.
***A(2)***: With foam insert. Electrodes unterminated extended along the Z-axis and grouped into two bundles: *Strip*, *Grid*, and *Depth 2* with 90 cm lead extensions and *Depth 1* and *Depth 3* without lead extensions.
***A(3)***: With foam insert. Electrodes extended along the Z-axis grouped into two bundles: *Strip*, *Grid*, and *Depth 2* with 90 cm lead extensions and connected to the EEG DAS (terminated), and *Depth 1* and *Depth 3* without lead extensions and unterminated.

The sequence of temperature measurements, with configuration, RF exposure sequence and manipulations, in *Experiment 1* are shown in Table 1.

**TABLE 1:** Experiment 1 configurations, manipulations and RF exposure data.

**Measurement #**	**Electrode configuration**	**Temperature probe locations**	**Other manipulations relative to previous measurement**	**RF exposure**			
				**Sequence type**	**SAR head (W/Kg) (% allowed maximum)**	**SAR whole-body (W/Kg) (% allowed maximum)**	**B_1_(μT) (% allowed maximum)**
1.1	*A(1)*	Ref, D2, D3, G	–	TSE*	3.2 (100)	0.8 (34)	4.6 (67)
1.2	*A(1)*	Ref, D2, D3, G	None	EPI	0.1 (3)	0 (1)	0.9 (12)
1.3	*A(1)*	Ref, D2, D3, G	None	TSE*	3.2 (100)	0.8 (40)	4.7 (67)
1.4	*A(1)*	Ref, D2, D3, G	None	EPI	0.1 (3)	0 (1)	0.9 (12)
1.5	*A(2)*	Ref, D2, D3, G	Table out, cable repositioning and table in	TSE	2.6 (82)	0.7 (33)	4.1 (58)
1.6	*A(2)*	Ref, D2, D3, G	None	TSE*	3.2 (100)	0.8 (34)	4.6 (65)
1.7	*A(2)*	Ref, D2, D3, G	Table out, cable repositioning and table in	TSE	3.1 (98)	0.8 (39)	4.5 (64)
1.8	*A(3)*	Ref, D2, D3, G	None	TSE	2.6 (82)	0.7 (33)	4.1 (58)
1.9	*A(3)*	Ref, D2, D3, G	None	EPI*	0.1 (3)	0 (1)	0.9 (12)

The exposure duration was 6 min 9 s*. Measurements 1.1 and 1.3, and 1.5, 1.6, and 1.7, respectively, constitute two sets of repeat measurements for configurations *A(1)* and *A(2)*, respectively. TSE, turbo spin echo; EPI, echo-planar imaging; Ref, reference; D2, *Depth 2*; D3, *Depth 3*; G, *Grid*. *Measurement shortened to 4:37 for measurements 1.1, 1.3, 1.6, and 1.9, due to lack of appreciable heating and scanner time access limitations for this experiment.

#### Experiment 2

This experiment constitutes an elaboration of *Experiment 1*, designed to explore the heating that results from scenarios that deviate more from our protocol (therefore akin to fault conditions), in particular in relation to the placement of the leads relative to the scanner’s central axis by not using the foam insert; also it provided additional (inter-session) reproducibility data.

**FIGURE 3:**
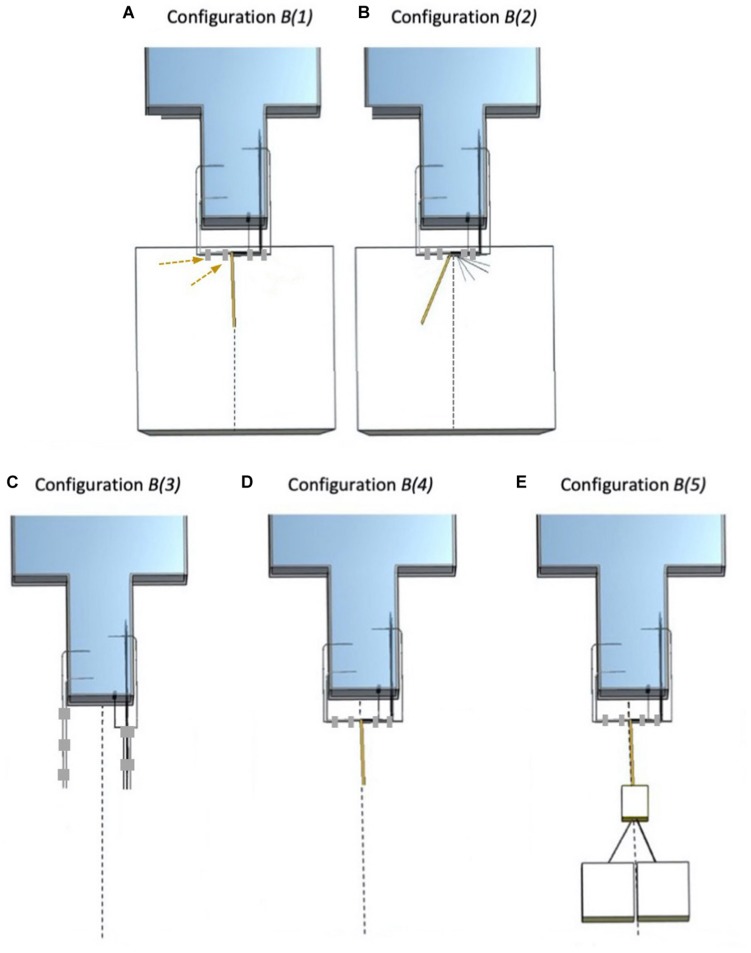
Electrode configurations B (*Experiment 2*). The dashed line represents the scanner’s central *Z*-axis. **(A)** Configuration *B(1)*: *Strip*, *Grid*, and *Depth 2* bundled together (right bundle), connected to 90 cm lead extensions and placed on top of the foam insert. *Depth 1* and *Depth 3* bundled together (left bundle) and unterminated with lead tails on top of the foam insert. The adhesive tape (gray) used to create the left bundle is highlighted with the dashed arrows. **(B)** Configuration *B(2)*: *Strip*, *Grid*, and *Depth 2* separated from each other and separated from *Depth 1* and *Depth 3. Depth 1* and *Depth 3* bundled together, connected to lead extensions (unterminated) and positioned a little away from the central Z-axis on top of the foam insert. **(C)** Configuration *B(3)*: *Strip*, *Grid*, *Depth 2*, *Depth 1*, and *Depth 3* leads without extensions, separated and unterminated; no foam insert. **(D)** Configuration *B(4)*: *Strip*, *Grid*, *Depth 2*, *Depth 1*, and *Depth 3* bundled together with *Depth 1* and *Depth 3* connected to the extensions (unterminated); no foam insert. **(E)** Configuration *B(5)*: *Strip*, *Grid*, *Depth 2*, *Depth 1*, and *Depth 3* bundled together, with *Depth 1* and *Depth 3* connected to the lead extensions and to the EEG DAS (extensions + terminated); no foam insert (i.e., EEG DAS placed on the bottom of the scanner bore).

The following five electrode configurations were studied in *Experiment 2* (see Figure 3):

***B(1)***: A repetition of *A(2)* (*Experiment 1*).
***B(2)***: With foam insert. Electrodes unterminated and placed away from the Z-axis. Grouped into two bundles: *Strip*, *Grid*, and *Depth 2* without lead extensions separated from each other, and *Depth 1* and *Depth 3* connected to the lead extensions.
***B(3)***: Without foam insert. No lead extensions. Unterminated. All electrode leads separated from each other.
***B(4)***: Without foam insert. Electrodes unterminated with *Depth 1* and *Depth 3* with lead extensions bundled together and *Strip*, *Grid*, and *Depth 2* without lead extensions separated from each other and from the other two.
***B(5)***: Without foam insert. Electrodes *Depth 1* and *Depth 3* bundled together and connected to the lead extensions and the EEG DAS (terminated), and *Strip*, *Grid*, and *Depth 2* without lead extensions separated from each other and from the other two.

The sequence of measurements of heating with the specified icEEG leads configurations and the applied MRI sequence in *Experiment 2* are shown in Table 2.

**TABLE 2:** Experiment 2 configurations, manipulations, and RF exposure data.

**Measurement #**	**Electrode configuration**	**Temperature probe locations**	**Other manipulations relative to previous measurement**	**RF exposure**			
				**Sequence type**	**SAR head (W/Kg) (% allowed maximum)**	**SAR whole-body (W/Kg) (% allowed maximum)**	**B_1_ (μT) (% allowed maximum)**
2.1	*B(1)*	Ref, D1, G, D3	–	TSE	3.2 (99)	0.6 (28)	4.5 (64)
2.2	*B(1)*	Ref, D1, G, D2	None	TSE	3.2 (99)	0.6 (28)	4.5 (64)
2.3	*B(2)*	Ref, D1, G, D2	Table out, cable repositioning and table in	TSE	3.2 (99)	0.6 (28)	4.5 (64)
2.4	*B(2)*	Ref, D1, G, D3	None	TSE	3.2 (99)	0.6 (28)	4.5 (64)
2.5	*B(3)*	Ref, D1, G, D3	Table out, cable repositioning and table in	TSE	3.2 (99)	0.6 (28)	4.5 (64)
2.6	*B(3)*	Ref, D1, G, D3	Table out, cable repositioning and table in	EPI	0.1 (3)	0 (1)	0.9 (12)
2.7	*B(4)*	Ref, D1, G, D3	Table out, cable repositioning and table in	TSE	3.2 (99)	0.6 (28)	4.5 (64)
2.8	*B(5)*	Ref, D1, G, D3	None	TSE	3.2 (99)	0.6 (28)	4.5 (64)

*Measurements 2.1 and 2.2, and 2.3 and 2.5, respectively, constitute two repeat measurements with different temperature probe locations (*Depth 1* and *Depth 2*, respectively; see section “Temperature Measurements”). The exposure duration was 6 min 9 s. TSE, turbo spin echo; EPI, echo-planar imaging; Ref, reference; D1, *Depth 1*; D2, *Depth 2*; D3, *Depth 3*; G, *Grid*.*

### MRI System and RF Exposure Sequences

The MRI scanner used in this investigation was a 1.5T Avanto (Siemens, Germany) in the Neuroradiology department of the National Hospital for Neurology and Neurosurgery (UCLH NHS Foundation Trust), London, United Kingdom; this is the scanner used for our icEEG-fMRI experiments on human subjects (Vulliemoz et al., 2011). All RF exposure in this work was performed using the scanner’s standard body RF-transmit coil.

In accordance with our previous tests (Carmichael et al., 2010) the nominal RF exposure duration was 6 min. Two MRI sequences were used: (1) turbo spin echo (TSE) to maximize heating (“worst case” scenario): TR = 2850 ms, TE = 92 ms, slice thickness/slice gap = 2.5/1.25 mm, FOV = 300 × 300 mm, in-plane resolution = 0.9 × 0.8 mm, BW = 125 Hz/pixel, FA = 180° and duration = 6 min 9 s. In Experiment 1, for four of the measurements, the exposure was reduced to 4 min 32 s due to lack of appreciable heating and scanner time access constraints (see Table 1); (2) EPI as used for icEEG-fMRI scanning (Carmichael et al., 2012), with the following parameters: TR = 4480 ms, TE = 50 ms, slice thickness/slice gap = 2.0/1.0 mm, FOV = 192 × 192 mm, in-plane resolution = 3.0 × 3.0 mm, BW 2298 Hz/pixel, FA 90° and duration = 6 min 4 s.

### Temperature Measurements

The temperature changes in the immediate vicinity of selected electrode contacts were monitored and recorded continuously using five fiber-optic sensors (model T1C-10-PP05 and model T1C-10-B05, Neoptix, Canada), connected to a 4-channel signal conditioner (Neoptix ReFlex—Neoptix, Canada). Based on prior experience we estimate the temperature measurement precision (standard deviation in the absence of heating) to be of the order of ±0.2°C. The temperature sensors were placed in five locations as follows: the tips of *Depth 1*, *Depth 2*, and *Depth 3*; contact number 48 of the *Grid* electrode which is located in the corner of the electrode and a reference location, at a depth of approximately 3 cm in the phantom gel, 10 cm away from all the electrodes corresponding roughly to the phantom’s neck area.

Because we were limited to four temperature channels simultaneously, in some tests we repeated the measurement with alternative temperature probes. In particular, following *Experiment 1* (see Table 3), in which we did not measure the temperature at *Depth 1* based on the results of our previous work (Carmichael et al., 2010) which suggested that the heating would be greatest at *Depth 3*. We tested this assumption in the first three measurements of *Experiment 2* (see Table 4). This demonstrated that the heating was greater at *Depth 1* than *Depth 2* (see Table 4), and therefore decided to record at *Depth 1* for the rest of that experiment.

**TABLE 3:** Experiment 1 results: maximum temperature increases.

**Measurement #**	**Type of sequence, configuration**	**Maximum temperature increases (°C)**			
		**Depth 2**	**Depth 3**	**Grid**	**Reference**
1.1	TSE, *A(1)*	0.6	0.3	0.1	0.1
1.2	EPI, *A(1)*	0.3	0.2	0.2	0.3
1.3	TSE, *A(1)*	0.7	0.2	0.3	0.3
1.4	EPI, *A(1)*	0.2	0.1	0	0.2
1.5	TSE, *A(2)*	0.3	0.2	0.4	0.4
1.6	TSE, *A(2)*	0.4	0.3	0.3	0.4
1.7	TSE, *A(2)*	0.2	0.2	0.3	0.5
1.8	TSE, *A(3)*	0.7	0.1	0.2	0.2
1.9	EPI, *A(3)*	0.3	0.2	0	0

**TABLE 4:** Experiment 2 results: maximum temperature increases.

**Measure ment #**	**Type of sequence, configuration**	**Maximum temperature increases (°C)**				
		**Depth 1**	**Depth 2**	**Depth 3**	**Grid**	**Reference**
2.1	TSE, *B(1)*	2.4	×	0.1	1	0.1
2.2	TSE, *B(1)*	2.1	0.2	×	0.5	0.4
2.3	TSE, *B(2)*	2.0	1.0	×	0.6	0.2
2.4	TSE, *B(2)*	1.7	×	0.2	0.5	0.3
2.5	TSE, *B(3)*	0.3	×	0.2	0.4	0.3
2.6	EPI, *B(3)*	0	×	0	0	0.1
2.7	TSE, *B(4)*	1.9	×	0.2	1	0.1
2.8	TSE, *B(5)*	4.5	×	0.1	1.1	0.1

*×, no measurement.*

## Results

### Experiment 1

**FIGURE 4:**
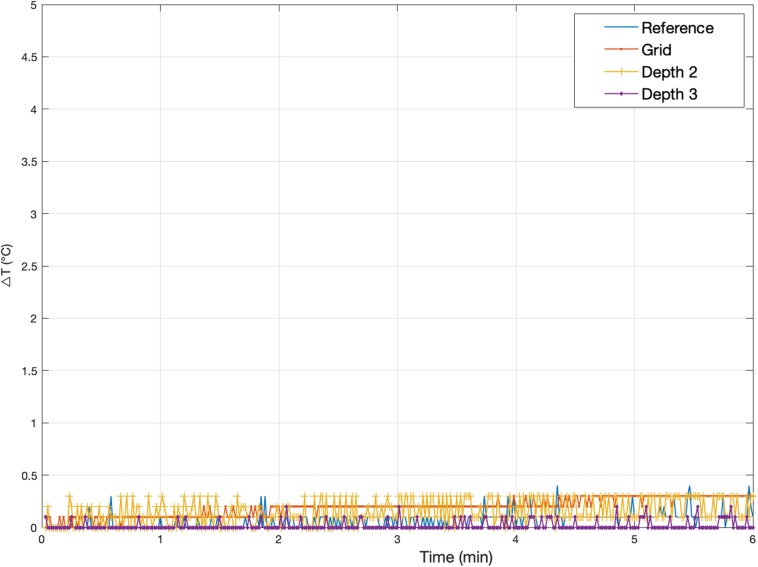
Representative temperature changes for *Experiment 1*. Measurement 1.5: Temperature changes at *Grid*, *Depth 1*, and *Depth 3* [electrode configuration *A(2)*] following exposure to a high-SAR (TSE) sequence starting at Time = 0. The plot also serves to illustrate the precision of the measurements, of the order of ±0.2°C. The peak temperature increases for every Experiment 1 measurement are reported in Table 3.

The maximum observed temperatures for all measurements in the presence of the foam insert across configurations *A(1)*–*A(3)* at every location can be found in Table 3. Figure 4 shows a typical temperature measurement series (measurement 1.1). The maximum temperature increase overall was 0.7°C at *Depth 2* for measurement 1.3, which was the electrode location of greatest heating for most measurements. In accordance with our expectations, the temperature increase values measured were greater for TSE than EPI; the maximum temperature changes for all EPI exposures were equal to, or below, 0.3°C, which is near the threshold of detectability (0.2°C), and comparable to the temperature increase at the reference position. Comparison of measurements 1.3 and 1.5–7 shows good reproducibility relative to phantom repositioning.

### Experiment 2

**FIGURE 5:**
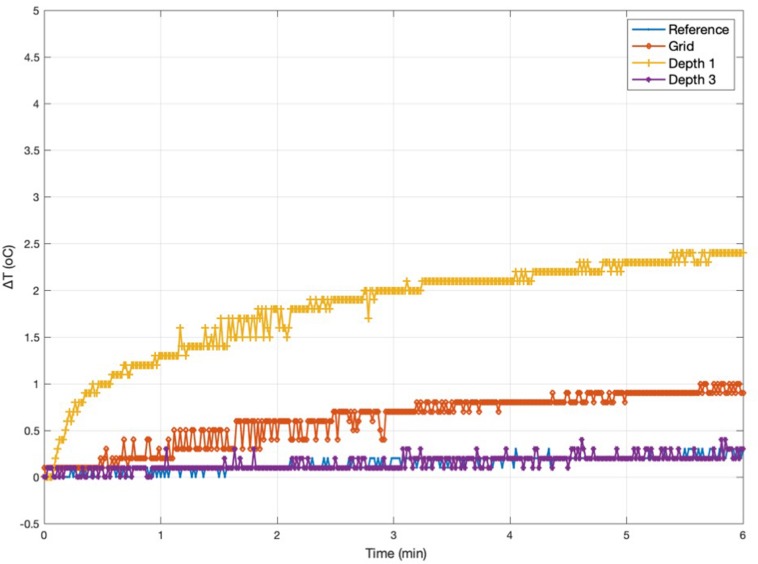
Representative temperature changes for *Experiment 2*. Measurement 2.1: temperature changes at *Grid*, *Depth 1*, and *Depth 3* [electrode configuration *B(1)*] following exposure to a high-SAR (TSE) sequence starting at Time = 0. The peak temperature increases for every Experiment 2 measurement are reported in Table 4.

Experiment 2 was performed one week after Experiment 1. The maximum observed temperature change values for all measurements are shown in Table 4. Figure 5 illustrates temperature changes for measurement 2.1. For the high-SAR sequence, the highest temperature increase recorded was 4.5°C, at electrode *Depth 1* (measurement 2.8). For the EPI sequence (measurement 2.6), the maximum temperature increase was negligible.

Measurements 2.1–2.4 are two sets of repeat measurements with the foam insert and lead extensions [configurations *B(1)* and *B(2)*, with extensions laid along, and away from the Z-axis, respectively]; these resulted in maximum temperature increases in the range 0.2–2.4°C for the depth electrodes, 0.5–1°C for the *Grid*.

For the remaining measurements, due to the limited number of channels of our temperature signal conditioning unit, and our wish to sample temperatures simultaneously (*Depth 1* not sampled in Experiment 1), we used the temperature probe at *Depth 1* instead of *Depth 2*, due to the higher temperatures observed at the former in measurements 2.2 and 2.3. After removing the lead extensions and foam insert (measurements 2.5 and 2.6), the maximum temperature increase dropped to 0.4°C across all electrodes (for the high-SAR sequence; negligible for EPI). Reconnecting the electrodes to the lead extensions (without foam insert; measurement 2.7) resulted in greater maximum temperature increases (1.9°C); connection to the digitization and amplification system (without foam insert; measurement 2.8) resulted in the maximum temperature increase of 4.5°C, at electrode *Depth 1*, the location of the greatest temperature increases in all measurements except 2.5.

## Discussion

We performed experiments to quantify the amount of heating induced in the immediate vicinity of a set of intracranial EEG electrodes by exposure to RF generated by a body transmit coil in a 1.5T MRI scanner. This work builds directly on our experience of acquiring concurrent icEEG-fMRI data using a quadrature head RF transmit coil in the MRI scanner (Vulliemoz et al., 2011; Chaudhary et al., 2016; Murta et al., 2016, 2017; Ridley et al., 2017; Sharma et al., 2019) and in particular the safety tests that made it possible (Carmichael et al., 2008) and associated scanning protocol (Carmichael et al., 2012). This protocol contains prescriptions on the choice of RF transmit coil, MR sequence, and the type, connection and positioning of the EEG wires and equipment, and relies to a large degree on the use of a scanner bore foam insert on which the EEG system can be placed precisely and consistently. In this work our objective was to confirm whether, based on the same protocol, the use of the body RF transmit coil instead of the head-only transmit coil in the same MRI scanner, would result in excessive heating. The electrodes were positioned inside a water-based gel phantom in a configuration that emulates a clinical scenario, in line with our previous tests (Carmichael et al., 2008) and subjected to trains of RF excitation pulses (low and high-SAR sequences) through the body RF transmit coil. We explored a range of electrode lead configurations: length, placement relative to the scanner’s central axis and termination; each a deviation from our previously defined protocol (Carmichael et al., 2012).

Current international guidelines recommend that MRI-induced heating should not cause temperature in the head to exceed 38°C, suggesting an allowable increase of ≤1°C (IEC, 2016).

In summary in this work, for the low-SAR (EPI) RF exposures prescribed in our protocol, the maximum observed temperature increase was 0.3°C across all tested configurations. This provides further evidence on the suitability of our established icEEG-fMRI protocol by extending its applicability to our 1.5T MRI scanner’s body RF-transmit coil. We assessed reproducibility by performing a number of repeated measurements within each experiment, for the same configuration, either by simply repeating the RF exposure (considering the high-SAR measurements only: measurements 1.1 and 1.3, 1.5 and 1.6, 2.1 and 2.2, 2.3 and 2.4) or repeating the RF exposure after moving the phantom assembly in and out of the scanner bore (measurement pair 1.6 and 1.7). Furthermore, taken together, measurements 1.5, 1.6, 1.7, 2.1, and 2.2 constitute repeated measurements for the same (intended) configuration [*A(2)* and *B(1)*] between scanning sessions (*Experiments 1* and *2*, which took place one week apart). The results of these comparisons (mean and standard deviation of inter-measurement difference across all locations: 0.0 and 0.2°C, respectively) give an indication of the good reproducibility of our measurements (Bland and Altman, 1995), and which combined with the reference temperature measurements, suggest a detection threshold of the order of 0.5°C.

To our knowledge there has been a single previous investigation of the safety of using body transmit coil for icEEG during fMRI at 1.5T: Ciumas et al. (2013) performed temperature assessments in a water-gel phantom and rabbit cadavers using an EPI sequence for depth electrodes in two orientations: axial and lateral, showing temperature increases in the range of 0.2 and 1.3°C. Previously, we investigated RF-induced heating for a body transmit coil at 3T for a set of electrodes placed similarly to the set up used in this work. For high-SAR exposures maximum temperature increases of 6.4°C at the grid electrode and 0.7°C at the depth electrodes were observed when the electrode leads and extensions were separated (“open circuit” configuration), while the temperature increases were lower when gathering the leads and extensions together in a bundle “short circuit” (Carmichael et al., 2008). In addition, when placing the leads and extensions close to the scanner bore, the maximum heating at the grid was found to be 2.9 and 6.9°C at the depth electrodes (Carmichael et al., 2012). Boucousis et al. (2012), working at 3T and using the body RF-transmit coil, observed a maximum temperature increase of 4.9°C when applying high-SAR and 0.5°C for low-SAR (fMRI) sequences. Both studies concluded that high-SAR sequences should be avoided when performing icEEG-fMRI; however, Boucousis et al. (2012) concluded that low-SAR sequences with the body transmit coil do not pose an unacceptable risk for the patients.

The purpose of performing heating tests with high-SAR RF exposures, even for the evaluation of scanning protocols that preclude them, is manifold: (1) assess the risks associated with worst case scenarios (operator error during application of the protocol); (2) ensure that conclusions reached based on low-SAR tests do not simply reflect temperature measurement sensitivity limitations; and (3) reflects the requirements specified in the standard guidelines (ASTM F-2182-02a; ASTM, 2011). In this study, for the high-SAR (TSE) exposures the maximum observed temperature increase was 4.5°C, for a configuration in which the wires and lead extensions were far from the scanner central axis (lying at the bottom of the scanner bore: no foam insert). This compares with a maximum temperature increases of 2.4°C across all configurations with the wires lying along the scanner’s Z-axis on top of our foam insert. We also note that this is much greater than the maximum increase of 0.7°C for the two configurations without lead extensions [*A(1)* and *B(3)*], thereby further confirming the important effect of lead length (Carmichael et al., 2010). Concerning the impact of circuit termination, which in our experiments tended to be associated with greater heating (measurements 1.8 vs 1.7 and 2.8 vs 2.7), this may result from this corresponding to a conductive loop, as opposed to capacitive effects between wires in close proximity. Furthermore, “avoiding loops” is the usual guidance when placing electrophysiological leads in the MR environment (Lemieux et al., 1997; Kainz et al., 2002; Balasubramanian et al., 2017). Importantly, the lead extensions and connection to the EEG DAS are necessary for the application of icEEG-fMRI. Therefore, while they can affect the risk level adversely, in particular the use of lead extensions, our aim was to demonstrate that the amount of heating created in the “with extensions and connected” condition was acceptable and in what conditions relative to other factors (positioning, sequence, and coil types).

The generalization of the conclusions that can be reached from our measurements is limited by numerous factors, including: the representativeness (and quality of fabrication) of the phantom and of the electrode configuration, the specific characteristics of the MRI scanner, and temperature measurement capability (spatial sampling, limited by the number of available temperature probes) and measurement error. While some of these, in particular the variety of possible electrode implantations used in clinical practice, may be particularly challenging we believe that this study is in line with previously published empirical work and furthermore reflects the ASTM standard-level of evidence. Similarly, in relation to spatial sampling of the temperature changes, our use of four temperature probes is also in line with many other recent studies (Boucousis et al., 2012; Ciumas et al., 2013; Jorge et al., 2015; Balasubramanian et al., 2017). We tried to mitigate this limitation using prior knowledge; for example, while in retrospect it might have been preferable to record the temperature at *Depth 1* instead of *Depth 2* in *Experiment 1*, we do not believe that this significantly alters our conclusions because *Experiment 2* was specifically conceived as a series of worst-case scenarios (this in contrast to *Experiment 1* which is effectively a feasibility test for the body transmit coil, based on our recommended configurations (Carmichael et al., 2012).

Therefore, the guidance that can be provided based on our results can be summarized as follows: icEEG-fMRI is feasible with acceptable risk on a 1.5T MRI scanner (TIM Avanto, Siemens, Erlangen, Germany) using the standard body RF transmit coil if the following restrictions are applied: the EEG leads are brought together as close as possible to the top of the head and placed exactly along the scanner’s central axis, and toward the back (head end) of the scanner, and connected to the EEG input box, itself placed on the scanner axis, which is connected to the EEG amplification units, also placed as close as possible to the scanner axis (this positioning can be facilitated by the use of a scanner foam insert (see Carmichael et al., 2012) and all scanning must be restricted to low-SAR gradient echo sequences.

## Conclusion

In summary, this study provides good evidence for the feasibility of simultaneous icEEG-fMRI utilizing the body transmit coil on the 1.5T Siemens Avanto MRI scanner at our center. Careful consideration of the positioning of the electrode leads and EEG amplification system and choice of sequences is crucial, and should follow our established protocol (Carmichael et al., 2012).

## Data Availability Statement

The datasets generated for this study are available on request to the corresponding author.

## Author Contributions

HH, AP, DC, and LL prepared the phantom for the experiment. HH and LL prepared and analyzed the data. HH wrote the manuscript. All authors contributed editorially.

## Conflict of Interest

The authors declare that the research was conducted in the absence of any commercial or financial relationships that could be construed as a potential conflict of interest.
